# Dual specificity phosphatase 1 as a non-invasive circulating biomarker candidate in preeclampsia

**DOI:** 10.3389/fendo.2025.1576240

**Published:** 2025-09-11

**Authors:** Jonatane Andrieu, Agathe Donet, Jean-François Cocallemen, Guillaume Charbonnier, Noémie Resseguier, Julien Paganini, Jean-Louis Mège, Soraya Mezouar, Florence Bretelle

**Affiliations:** ^1^ Aix-Marseille Univ, Centre National de la Recherche Scientifique, Établissement Français du Sang, Anthropologie bio-culturelle, Droit, Éthique et Santé, Marseille, France; ^2^ Aix-Marseille Univ, Institut Recherche Développement, Assistance Publique – Hôpitaux de Marseille, Microbe, Evolution, Phylogeny Infection, Marseille, France; ^3^ Department of Gynecology-Obstetric, La Conception Hospital, Marseille, France; ^4^ Department of Bioinformatics and Biostatistics, Xegen, Gemenos, France; ^5^ Aix-Marseille Univ, Assistance Publique – Hôpitaux de Marseille, La Timone Hospital, Department of Epidemiology and Health Economics, Clinical Research Unit, Direction of Health Research, Marseille, France; ^6^ Department of Immunology, La Conception Hospital, Marseille, France; ^7^ Faculty of Medical and Paramedical Sciences, Aix-Marseille University, Health Improvement Through Physical Exercise (HIPE) Human Lab, Marseille, France

**Keywords:** preeclampsia, placenta, hypertension, DUSP1, RNA-seq

## Abstract

**Introduction:**

Preeclampsia (PE) is a multisystem pregnancy complication. Factors pointing to a placental origin are the development of the pathology only during pregnancy, and its disappearance in the post-partum period.

**Methods:**

Here, we aim to identify early predictive biomarkers. Whole blood and serum samples were collected at the time of the first event of PE (V1) and same samples after remote delivery (30-60 postpartum days, V2). These two samples enabled investigation of PE markers found in V1 but absent in V2. To confirm that these candidates are associated with PE, an investigation of associated placental biopsy was also realized (J0).

**Results:**

Our study identified a specific signature of PE including five Gene Ontology clusters including “angiogenesis and differentiation”, “cell-cycle”, “cell-adhesion”, “inflammatory response” and “cellular metabolism”. DUSP1 (Dual Specificity Phosphatase 1) gene was found specifically modulated in PE. PE women have a higher concentration of DUSP1 in serum compared to healthy donors. Interesting, at a distance from childbirth (V2), DUSP1 finds a rate like control group showing its predictive interest as a promising predictive biomarker of PE.

**Discussion:**

The investigation of DUSP1 in a prospective study with a larger cohort, including the severity aspect of the disease, is necessary to confirm its value as a predictive biomarker in PE.

## Introduction

Preeclampsia (PE) is a progressive, multisystem pregnancy complication that affects 3%–5% of pregnancies, making it one of the major causes of maternal and fetal morbidity and mortality (1). PE is responsible for hematological complications and severe organ failure, particularly affecting the placenta, nervous system, liver, lungs, kidneys, and cardiovascular system (2, 3). Fetal complications include life-threatening complications such as intrauterine growth retardation, malformations, and induced prematurity (4). PE is a complex pathological process that originates at the maternal–fetal interface (5, 6). It is widely accepted that PE is a disease of maternal endothelium with placental origins. Supporting this theory is the observation that the pathology develops only during pregnancy and resolves in the postpartum period. 

Several early prognostic clinical indicators (e.g., mean arterial pressure) and ultrasonography markers (e.g., uterine artery pulsatility index) have been combined to diagnose PE. At the biological level, placental growth factor (PlGF) and pregnancy-associated plasma protein A (PAPP-A) have been proposed to predict the risk of preterm PE. With a positive predictive value of approximately 85%–90%, the Fetal Medicine Foundation (FMF) test was developed to assess the risk of early PE. This means that 10%–15% of FMF tests may yield a high-risk result but will not result in premature PE (1). Other studies have focused on trophoblastic cells, as placental cells, by examining their processes of migration and invasion (7). Markers such as programmed death-ligand 1 (PD-L1) (8) and angiopoïetine like 4 (ANGPTL4) (9) have been shown to significantly increase trophoblast invasion and migration in PE, and have also implicated the yes-associated protein (YAP)–Hippo trophoblast differentiation pathway (10). However, these factors only contribute to a better understanding of PE physiopathology.

There has been growing interest in early predictive biomarkers for PE. Effective predictive tests would facilitate early diagnosis, targeted monitoring, and prompt management, using biomarkers capable of identifying risk early in pregnancy (before 16 weeks) in women at high risk of clinical complications (11). The anti-angiogenic factor soluble fms-like tyrosine kinase 1 (sFlt-1), found in the placenta and measured in plasma and serum, has been proposed as a specific biomarker for the onset and severity of PE (12). Evaluation of the ratio of sFlt-1 to the pro-angiogenic factor PlGF was found to have a high negative predictive value (13) and can be used to predict the short-term absence of PE in women for whom the disease was previously suspected clinically. Unfortunately, its predictive value is strongly linked to the prevalence of the disease. Ongoing studies are focused on the selection of women for early intervention to prevent PE onset, particularly through acetylsalicylic acid prescription (14). The ASPRE trial showed that identification of at-risk women using a score that includes mean arterial pressure, uterine artery pulsatility index, and maternal serum PAPP-A and PlGF can reduce early PE (15, 16). However, the overall rate was not decreased, which encourages further studies on the identification of new tools or factors.

The aim of this study was to identify new early biomarkers of PE based on a transcriptional signature present at the time of the event, using both maternal peripheral blood and placental biopsy samples. The secondary objective was to evaluate the evolution of this signature’s expression during the progression of pregnancy, particularly at the time of delivery, using samples from maternal blood and placental tissue.

## Materials and methods

### Ethics statement

This single-center, prospective, longitudinal study was conducted in accordance with the Declaration of Helsinki and French laws on research involving humans. The study protocol was approved by an independent national ethics review board, “CPP Sud Mediterranean 1” (approval no. 2010-A00633-36). All pregnant women provided written informed consent. Participants were recruited at the gynecology–obstetrics departments of Hôpital de la Conception and Hôpital Nord (Marseille, France) between February 2019 and July 2020.

### Study participants and sample collection

The study included 10 pregnant women as controls and 10 pregnant women diagnosed with PE between 20 and 37 weeks of gestation (**Table 1**). Pregnant women with PE presented with arterial hypertension (systolic blood pressure greater than or equal to 140 mmHg and/or diastolic blood pressure greater than or equal to 90 mmHg) associated with proteinuria (positive urine dipstick or proteinuria greater than 0.3 g protein per 24 h). PE and control groups were matched for maternal age and gestational age at inclusion.

**Table 1:** Initial characteristics of the population at the time of inclusion.

Characteristics	Control (n=10) n (%)	Preeclampsia (n=10) n (%)	*p-value*
Pregnant women			
Age (years)	29.50 ± 4.45	31.20 ± 7.43	*0.54*
Geographical origin • Caucasian • African • Asian	7 (70) 3 (30) 0	9 (90) 1 (10) 0	*0.58*
BMI (Kg/m^2^)	23.70 ± 4.27	24.50 ± 3.98	*0.68*
Smoking status • Absence • Active (>10 cig/day)	9 (90) 1 (10)	10 (100) 0 (0)	–
Obstetrical characteristics			
Gestational age at diagnosis (weeks)	31.4 ± 4.30	29.5 ± 3.13	*0.29*
Conception type • Spontaneous pregnancy • Induced IVF pregnancy	10 (100) 0 (0)	8 (80) 2 (20)	*0.47*
Delivery route • Vaginal delivery • Caesarean section	8 (80) 2 (20)	0 (0) 10 (100)	*0.0007*
Maternal complications • Absence • Presence - Uncontrolled hypertension - Proteinuria >6g/day - Acute renal failure - HELLP syndrome	10 (100) 0 (0) 0 (0) 0 (0) 0 (0) 0 (0)	6 (60) 4 (40) 3 (30) 2 (20) 1 (10) 2 (20)	*0.0867*
Fetal outcome			
Gestational age at birth (weeks)	39.5 ± 1.13	30.1 ± 3.1	*<0.001*
Days between inclusion and delivery	61.6 ± 31.25	3.3 ± 3.05	*<0.001*
Fetal growth • Eutrophic fetus • Intrauterine growth retardation	10 (100) 0 (0)	7 (70) 3 (30)	*0.21*
Fetal Doppler • Normal fetal Doppler • Doppler anomalies	10 (100) 0 (0)	7 (70) 3 (30)	*0.21*
Neonatal complications • Absence • Presence - Fetal growth restriction - Respiratory distress - Neonatal death	8 (80) 2 (20) 0 (0) 2 (20) 0 (0)	2 (20) 8 (80) 4 (40) 3 (30) 1 (10)	*0.02*
Birth weight (g)	3174.4 ± 281	1203.5 ± 611.1	*<0.001*

BMI, body mass index; HELLP syndrome, syndrome of hemolysis, elevated liver enzymes, and low platelet count.

Clinical parameters recorded included maternal age, geographic origin, body mass index (kg/m²), and obstetrical characteristics (gestational age, parity, spontaneous or induced pregnancy, and any pregnancy-related complications). Detailed fetal outcomes were monitored, including ultrasound findings, fetal heart rate analysis, and neonatal data.

Total blood samples (PAXgene tubes, PreAnalytiX) were collected at the time of PE diagnosis (and at matched gestational age for controls) and again 4–6 weeks postpartum (**Supplementary Figure S1**). PAXgene tubes were stored at 4°C for 24 h, then frozen at −20°C for 24 h before permanent storage at −80°C. A placental biopsy was also performed at the time of delivery for all participants. Each biopsy consisted of a macroscopically selected placental area of 2x2 cm including both chorionic and basal membranes. Biopsies were preserved in RNA*later* (Thermo Fisher Scientific) for 24 h at 4°C, then frozen for 24 h at −20°C, and finally stored at −80°C.

### RNA extraction

Total RNA from whole blood samples was extracted using the PAXgene Blood RNA Kit (Qiagen) according to the manufacturer’s instructions. Briefly, total blood was lysed using proteinase K, and nucleic acids were precipitated by ethanol. DNA was digested with RNase-free DNase I for 15 min at room temperature. Total RNA was eluted and incubated at 65°C for 5 min before being stored at -80°C.

Total RNA from placental biopsies was extracted using the RNeasy Mini Kit according to the procedure recommended by the manufacturer (Qiagen). After dissolution of placental tissue in RLT buffer with (RLT)-β-mercapto-ethanol, nucleic acids were precipitated with ethanol. DNA digestion was performed with RNase-free DNase I for 15 min at room temperature. Total RNA was eluted and stored at −80°C.

The quality and quantity of extracted RNA were evaluated using the Bioanalyzer 2100 (Agilent Technologies) and a NanoDrop Spectrophotometer (Nanodrop Technologies).

### RNA-sequencing and data processing

Reads were aligned and quantified using STAR (https://doi.org/10.1093/bioinformatics/bts635) on the hg19 genome assembly with GENCODE v19 annotations. The raw gene count table was variance-stabilized and reduced into principal components and uniform manifold approximation and projection (UMAP) for quality control. The raw count table was also used to perform differential expression analysis (DEA) using the Deseq2 framework (17), with apeglm shrinkage applied to the log_2_ fold change (18). Individual DEA results were compiled into integration plots, retaining genes that were significant in at least one design based on a Benjamini–Hochberg adjusted p-value <0.05 in at least one design. Data from RNASeq data analysis were submitted on the GEO data collection (GSE262147). Gene expression changes (up- or downregulation) were evaluated relative to control samples.

### Quantitative reverse transcription-polymerase chain reaction

Reverse transcription of isolated RNA was performed using the Moloney murine leukemia virus reverse transcriptase kit (Life Technologies) and oligo(dT) primers. Gene expression was evaluated using real-time qPCR with the Smart SYBR Green Fast Master Kit (Roche Diagnostics) and specific primers (**Supplementary Table S1**). qPCRs reactions were performed using a CFX Touch Real-Time PCR Detection System (Bio-Rad). Results were normalized to the expression of the *ACTB* housekeeping gene and are expressed as relative quantity (RQ) using the 2^-ΔCt^ with ΔCt = Ct_Target_ – Ct*_ACTB_* as previously described (19).

### Immunoassays

FLT1 (fms related receptor tyrosine kinase 1) and DUSP1 (Dual Specificity Phosphatase 1) levels were quantified in serum from study population with appropriate ELISA (enzyme-linked immunosorbent assay) according to the manufacturer’s instructions (Antibodies). The sensitivity was 6.99 pg/ml for FLT1 and 9.4 pg/ml for DUSP1.

### Protein interactome

The protein interactome between DUSP1 and FLT1 was generated using the STRING functional association networks protein software.

### Statistical analysis

Descriptive statistics of the initial characteristics of the population were carried out using R software version 3.6.1. Quantitative variables were described using the mean and standard error of the mean (SEM). Qualitative variables were described using percentages and p-values. Categorical variables were compared using the Chi-square test or Fisher’s exact test, as appropriate. The alpha risk was defined at 5%. Statistical analysis of gene signatures was performed using GraphPad Prism 6 (Graphpad Software Inc.). Gene expression was analyzed using the one-way ANOVA (analysis of variance) test and Tukey’s multiple comparisons test. Values represent the mean ± SEM. The limit of significance was set at *p<0.05*.

## Results

### Study design

We conducted a prospective, longitudinal study to investigate novel biomarkers for PE diagnosis. Ten patients with PE were included during the study period at a university medical center. Ten pregnant women with normal pregnancies and no significant medical history were matched as controls to the PE patients based on maternal age and gestational age at the time of PE diagnosis.

The study design is shown in **Supplementary Figure S1**. Whole blood and serum samples were collected at the time of the first PE event (V1), and the same types of samples were collected after remote delivery (30 to 60 postpartum days, V2). These two samples enabled the investigation of PE markers found in V1 but absent in V2. To confirm that these biomarkers were associated with PE, placental biopsies collected after delivery were also analyzed (J0).

We first focused on the study population at the time of inclusion. As illustrated in **Table 1**, maternal age (years) at diagnosis was comparable between cases and controls, 31 ± 7.43 and 29.5 ± 4.45, respectively (*p=0.54*). Gestational age (weeks) at diagnosis showed no significant difference between the two groups: 29.54 ± 3.13 in PE and 31.38 ± 4.30 in controls (*p=0.29*). No significant differences were observed for body mass index and smoking.

Considering pregnancy outcomes in the two groups (**Table 1**), as expected, gestational age at delivery was significantly earlier in the PE group (30.07 ± 3.12) than in the control group (39.52 ± 1.13) (*p<0.001*). The time between inclusion and delivery (days) was significantly shorter in the PE group (3.3 ± 3.05) than in the control group (61.6 ± 31.25) (*p<0.001*). Patients with PE delivered by cesarean section in 100% of cases, compared to 20% in the control group (*p* = *0.0007*). Serious maternal complications were observed in the PE group, such as uncontrolled hypertension (30%), heavy proteinuria (20%), acute renal failure (10%), and HELLP syndrome (20%). However, no significant differences were observed between the two groups (*p* = *0.0867*).

Similarly, there were also significant differences in neonatal outcomes. Neonatal weight (g) was significantly lower in the PE group (1,203.5 ± 611.1) than in the control group (3,174.4 ± 28) (*p<0.001*). Neonatal complications were significantly increased in the PE group (80% vs. 20%, *p=0.02*). In our cohort, they mainly consisted of severe sepsis (40%) and respiratory distress (30%), as well as one case of neonatal death.

### Preeclampsia RNA profile

**Figure 1:**
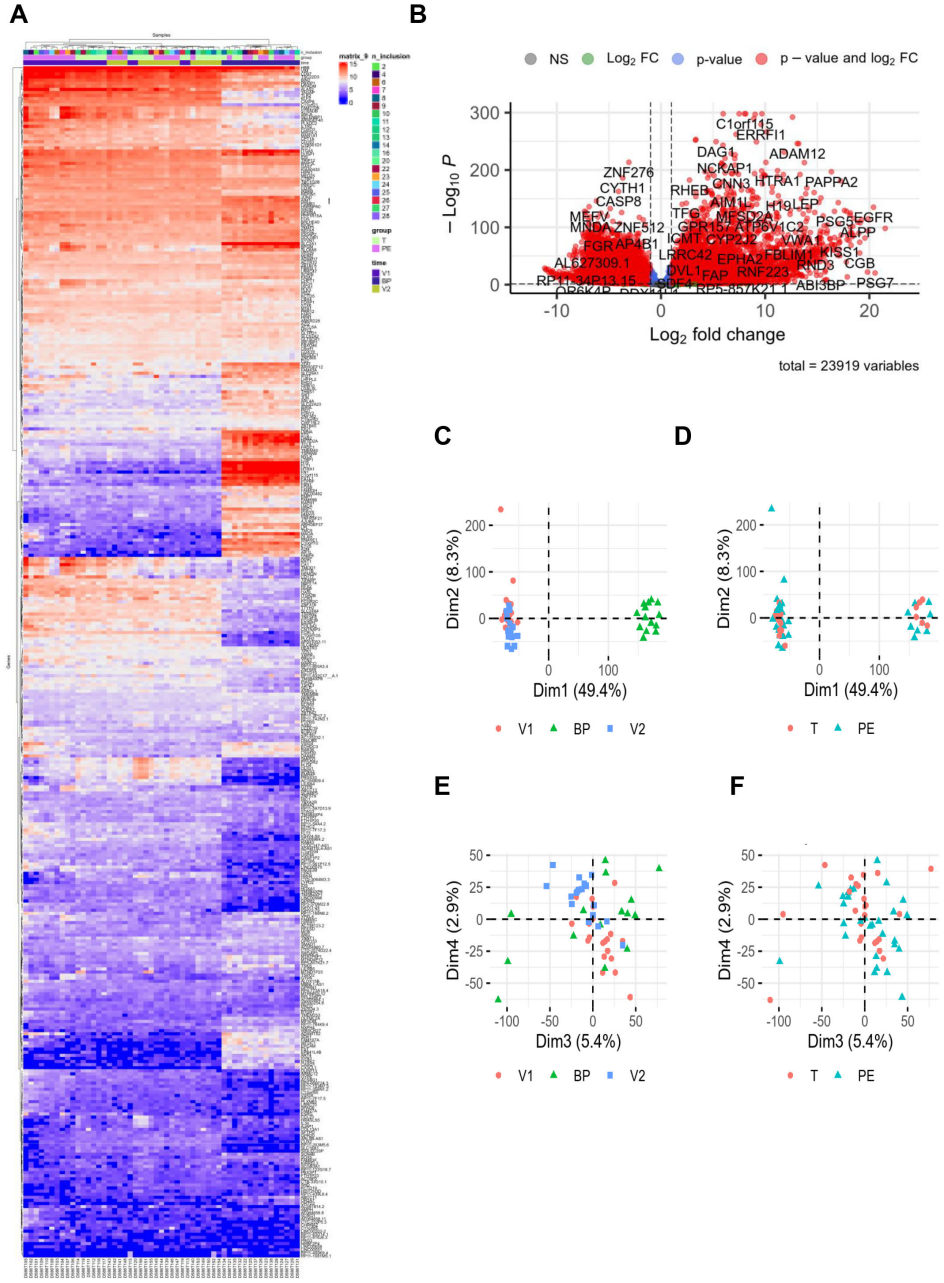
RNAseq data. RNA sequencing was performed with 24 healthy controls (C, 10V1, 8V2, 6BP) and 29 preeclamptic women (PE, 10V1, 10V2, 9BP). **(A)** Hierarchical clustering and **(B)** volcano plot highlighted modulated genes from RNA-seq data analysis, revealing 23,919 differentially expressed genes (fold change >2 and false discovery rate [FDR] <0.05). For the hierarchical clustering, “n_inclusions” corresponds to the number assigned to included patients in the cohort. The groups are T = control (light green square) and PE = preeclampsia (pink square). “Time” corresponds to the three types of sampling: V1 = first blood sampling (purple square), BP = placental biopsy performed on the day of delivery (dark blue square), and V2 = *post-partum* blood sampling (green square). **(C–F)** Principal component analysis illustrated the distribution of the investigated groups (V1, BP, and V2). BP, biopsy from placenta; FC, fold change; T, control group.

After raw data normalization, differences between samples from pregnant women with PE and healthy donors were visualized in **Figure 1**. The hierarchical clustering heatmap showed that placental samples clustered separately from whole blood samples (**Figure 1A**). RNA-seq analysis revealed 23,919 differentially expressed genes (fold change >2 and false discovery rate (FDR) < 0.05) with sufficient variance for statistical analysis using DESeq2, as illustrated in the volcano plot (**Figure 1B**). Principal component analysis demonstrated contrasts among the two investigated groups regarding the sample type (**Figure 1C**) but not by study group (PE vs. control) (**Figure 1D**). When the sample type variable was excluded, no clear grouping emerged by study group among individuals (**Figures 1E, F**).

**Figure 2:**
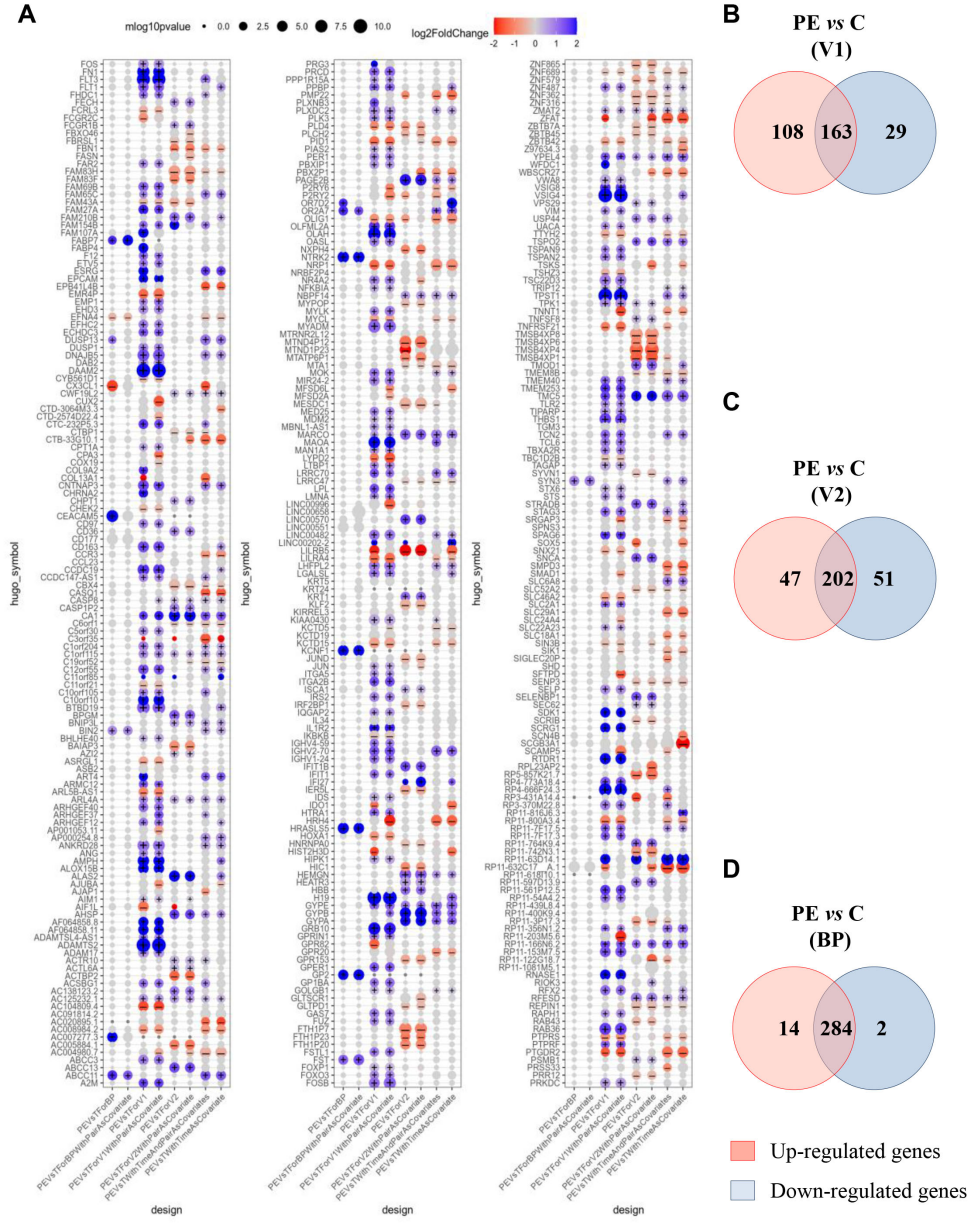
Modulated genes associated with preeclampsia. **(A)** Clusters show 300 modulated genes obtained after adjustment and selection based on variance and p<0.05, according to the Benjamini–Hochberg method. **(B–D)** Venn diagrams illustrated up- and down-modulated genes in preeclampsia (PE) *versus* control group for **(B)** V1, **(C)** V2 and **(D)**. BP, biopsy from placenta. Each intersection shows the number of genes that are neither up- nor down-regulated. (+) and (–) indicate p-value <0.05 and absolute value of log2FoldChange > 0.1.

**Figure 3:**
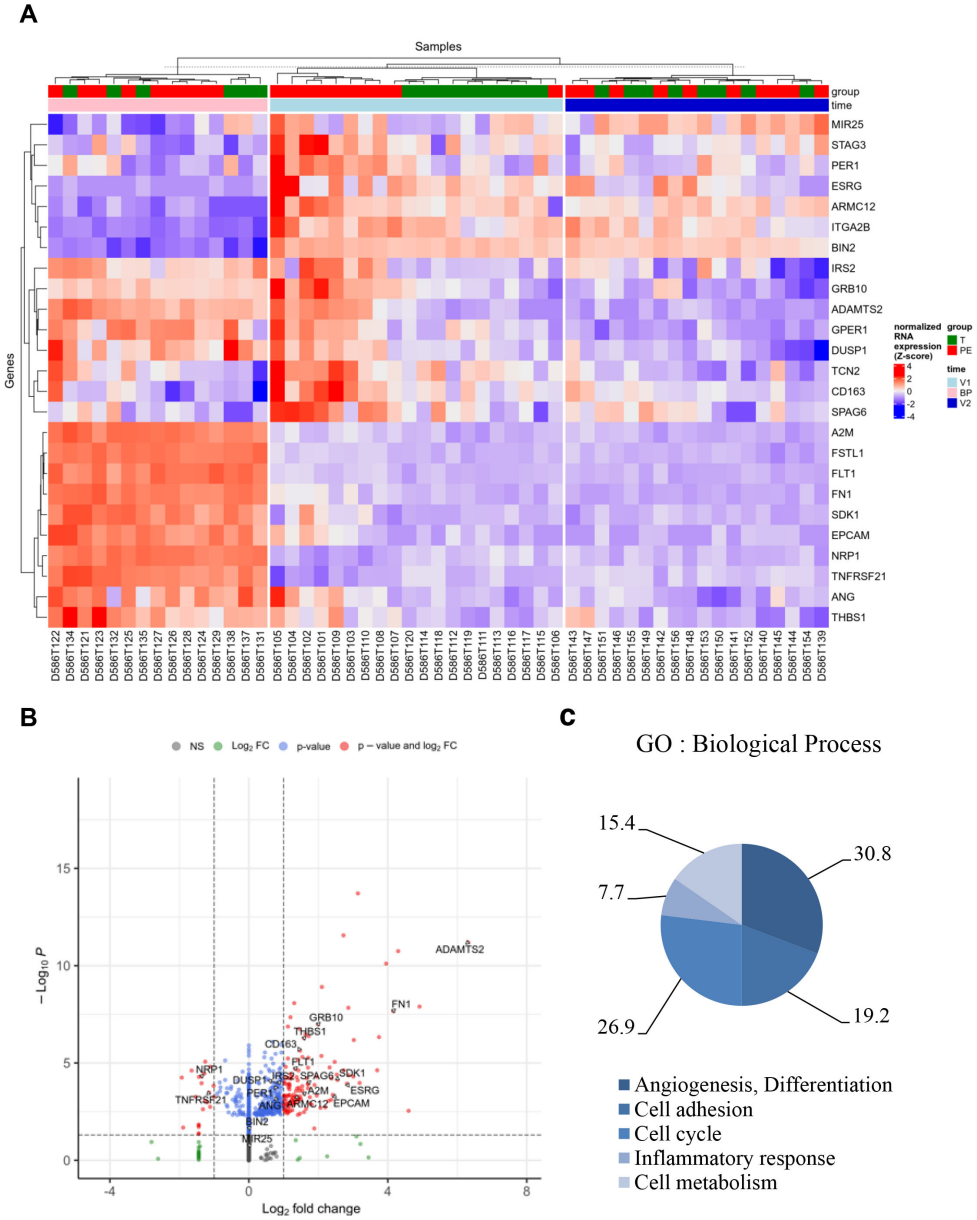
Specific genes associated with preeclampsia. Based on the selection of 300 genes modulated in V1, absent in V2, and present in the placental biopsy (BP) for the PE group compared to the control (T) group, 25 genes were identified as a specific signature of PE. **(A)** Hierarchical clustering and **(B)** volcano plot illustrated the 25 modulated genes. **(C)** Graph illustrating the Gene Ontology (GO) analysis based on “Biological Process,” including the percentage of genes associated with “angiogenesis and differentiation,” “cell cycle,” “cell adhesion,” “inflammatory response,” and “cellular metabolism.” FC, fold change.

We next investigated gene modulation between PE and control groups at each of the three time points: V1, BP, and V2. Differential expression analysis was adjusted for time as a covariate. After filtering for variance and significance (p<0.05), 300 genes were identified as significantly modulated based on Benjamini–Hochberg adjusted p-value <0.05 in at least one comparison. When focusing on the model adjusted for time, 27% of these genes (81) were upregulated and 20% (61) were downregulated (**Figure 2A**). Notably, at the time of first inclusion (V1), corresponding to the initial PE event, 108 genes were found to be upregulated in whole blood samples from the PE group compared to controls (**Figure 2B**). A similarly high number of upregulated genes was observed in the transcriptional signature of placental biopsies (**Figure 2D**). In contrast, at V2—corresponding to the postpartum sample—upregulated and downregulated gene counts were more balanced. The aim of this study was to determine relevant biomarkers that might reflect the pathophysiological mechanisms underlying PE. We therefore focused on genes that were up- or down-regulated in whole blood at V1, absent at V2, and concurrently expressed in placenta samples from PE patients but not controls. Under these conditions, 25 genes were identified as a specific PE signature, as shown in the hierarchical clustering (**Figure 3A**) and volcano plot (**Figure 3B**).

Gene Ontology (GO) analysis of “Biological Process” terms revealed five GO clusters. In decreasing order, 30.8% of genes were associated with “angiogenesis and differentiation,” 26.9% with “cell cycle,” 19.2% with “cell adhesion,” 15.4% with “cellular metabolism,” and 7.7% with “inflammatory response” (**Figure 3C** and **Supplementary Table S2**).

### Identification of a specific signature for preeclampsia

**Figure 4:**
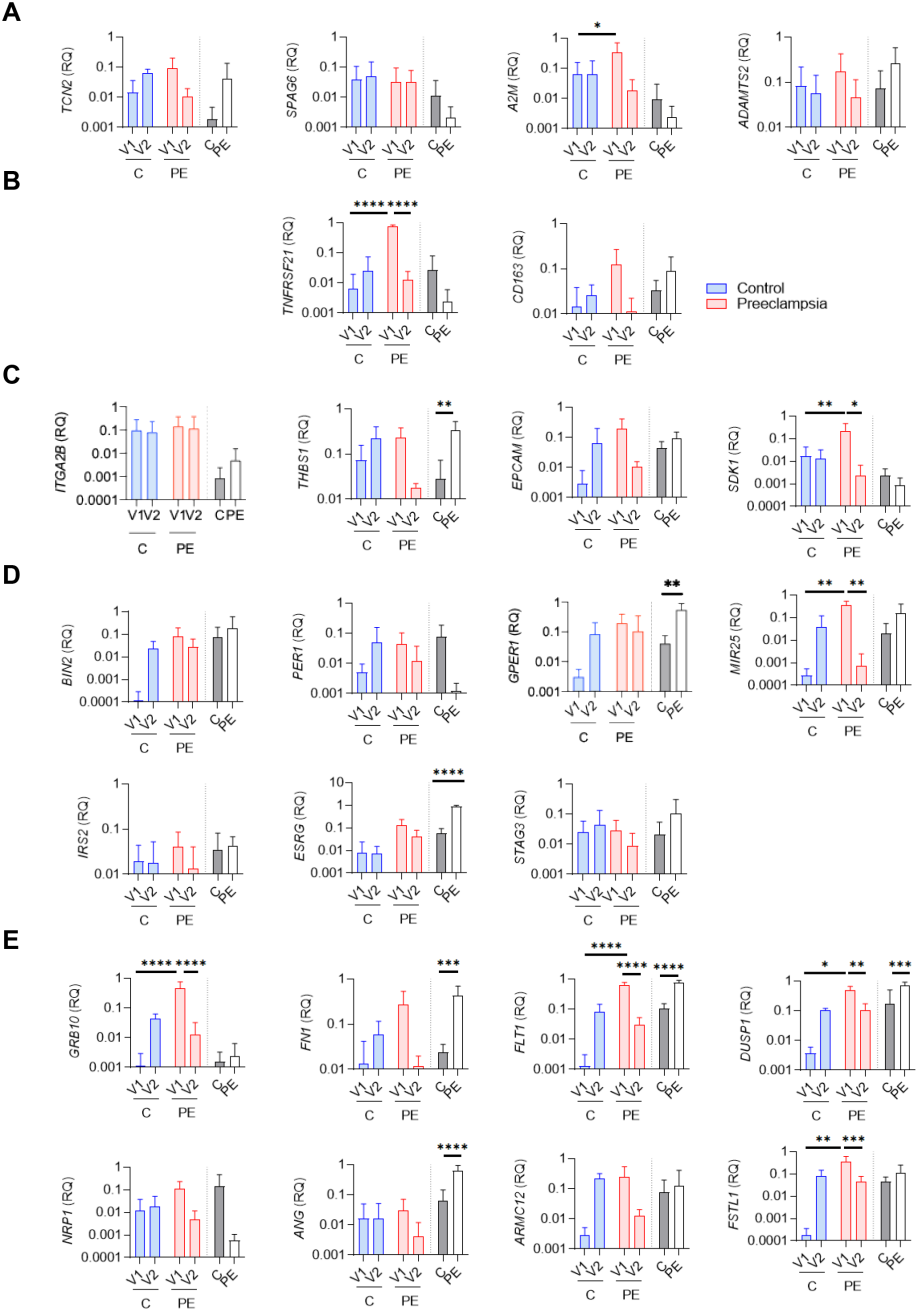
qRTPCR evaluation of specific genes associated with preeclampsia. Relative quantity evaluation of genes involved in **(A)** “cellular metabolism,” **(B)** “inflammatory response,” **(C)** “cell adhesion,” **(D)** “cell cycle,” and **(E)** “angiogenesis and differentiation” pathways. Modulated genes were obtained after qRTPCR experiments using whole blood (V1, V2) and biopsy from placenta (BP) from six healthy controls **(C)** and six preeclamptic women (PE), shown with gray and white bars, respectively. Data values represent the mean ± standard error of the mean (SEM); experiments were carried out in triplicate. Statistical analysis was performed with one-way ANOVA (analysis of variance) and Tukey’s multiple comparison test. *p ≤ 0.05, **p ≤ 0.01, ***p ≤ 0.001 and ****p≤ 0.0001.

Genes identified were next evaluated using quantitative reverse transcription–polymerase chain reaction (qRT-PCR) (**Figure 4**). Among the genes associated with “cellular metabolism,” only *A2M* showed a significant difference between V1 and V2 in the PE group (*p* = *0.0236*) (**Figure 4A**). No significant differences were observed for *TCN2, SPAG6*, and *ADAMTS2*.

Within the “inflammatory response” cluster, only *TNFRSF21* was significantly increased at V1 in the PE group compared to the control group (*p<0.0001*), and a significant decrease was observed in the PE group between V1 and V2 (*p<0.0001*) (**Figure 4B**). No differences were found for *CD163*.

Among the four genes associated with the “cell adhesion” cluster (*ITGA2B*, *THBS1*, *EPCAM*, *SDK1*), two (*THBS1* and *SDK1*) were differentially modulated between the PE and control groups (**Figure 4C**). *THBS1* was significantly increased in the PE group at the placental level (*p=0.0073*), although no statistical difference was observed at the blood level. *SDK1* was significantly increased at V1 in PE compared to the control group (*p=0.0099*), and showed a significant decrease between V1 and V2 in the PE group (*p=0.0267*) (**Figure 4C**).

Among the seven genes associated with the “cell cycle” cluster (*BIN2*, *PER1*, *MIR25*, *IRS2*, *ESRG*, *STAG3, GPER1*), three were differentially modulated between the PE and control groups (**Figure 4D**). In whole blood, *MIR25* was significantly increased at V1 in PE compared to the control group (*p* = *0.0012*) and for the PE group, a significant decrease was observed between V1 and V2 (*p=0.044*) (**Figure 4D**). At the placental level, *ESRG* and *GPER1* were significantly increased in the PE group compared to the control (*p<0.0001* and *p=0.0017*, respectively).

Finally, we identified eight modulated genes associated with the “angiogenesis and differentiation” cluster (*GRB10, FN1, FLT1, DUSP1, NRP1, ANG, ARMC12, FSTL1*) (**Figure 4E**). Among them, six genes were found differentially modulated between the investigated groups (*FN1, FLT1, ANG, GRB10, FSTL1, DUSP1*). *FN1* and *ANG* were significantly increased in placental biopsies from PE patients compared to controls (*p=0.0007* and *p<0.0001*, respectively). *FSLT1* and *GRB10* were significantly increased at V1 in PE compared to the control group (*p=0.0007* and *p<0.0001*, respectively), and both showed significant decreases between V1 and V2 in the PE group (*p<0.0001* and *p=0.007*, respectively). Interestingly, *FLT1*, a well-established biomarker in PE (13), also showed consistent modulation in our study. *FLT1* was significantly increased at V1 in the PE group compared to the control group (*p<0.0001*) and significantly decreased at V2 in the PE group compared to V1 (*p<0.0001*). At the placental level, FLT1 was also significantly overexpressed in PE patients compared to controls (*p<0.0001*), further confirming its relevance in PE pathophysiology (13, 20–22)Among all the investigated genes, *DUSP1* showed the same state of significant expression modulation as *FLT1*: (1) significantly increased at V1 in PE compared to controls (*p=0.0185*); (2) significantly decreased at V2 in PE compared to V1 (*p=0.0011*); and (3) significantly increased at the placental level in PE compared to controls (*p* = *0.0006*). Taken together, our findings highlight *DUSP1* as a promising gene of interest in PE.

### *DUSP1* modulation in preeclampsia

**Figure 5:**
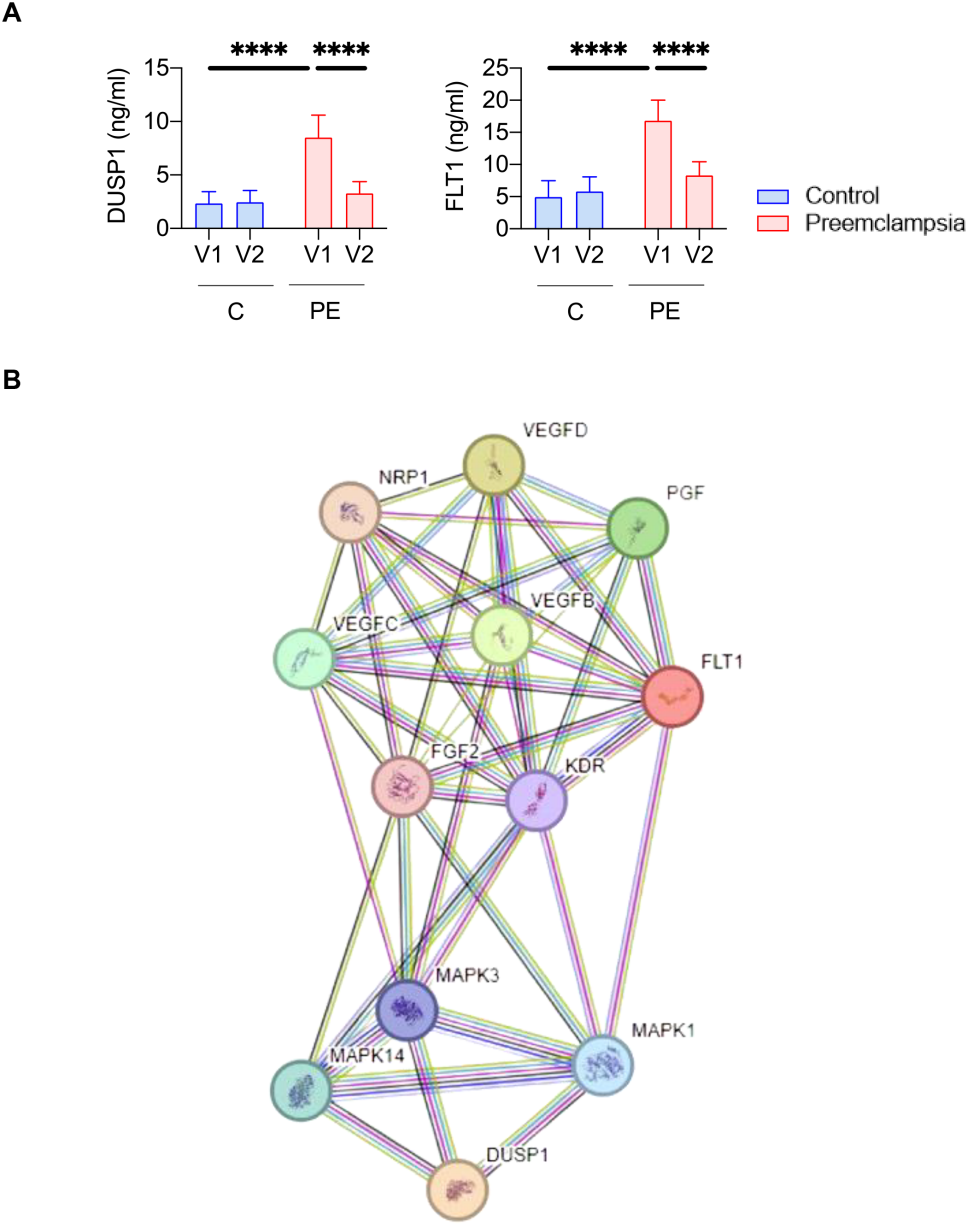
DUSP1 represents a biomarker candidate of preeclampsia. **(A)** Quantification of DUSP1 and FLT1 protein levels by immunoassay in serum samples (V1 and V2) from nine controls (C) and nine preeclamptic women (PE). Statistical analysis was performed using one-way ANOVA (analysis of variance) and Tukey’s multiple comparisons test. ****p≤ 0.0001.. **(B)** Protein pathways linked to DUSP1.

We next evaluated levels of DUSP1 in serum samples using immunoassays. As illustrated in **Figure 5A**, DUSP1 was barely detected in healthy donor serum during pregnancy (V1) or postpartum (V2). Interestingly, pregnant women with PE showed significantly higher concentrations of DUSP1 at V1 compared to controls (*p<0.0001*), suggesting that DUSP1 could be an interesting biomarker. Focusing on the PE group, we found that DUSP1 levels decreased after childbirth; at V2, levels were similar to those observed in the control group (*p<0.0001*). A similar modulation pattern was observed for FLT1 concentrations, which were significantly elevated in PE donors compared to healthy donors at V1, then decreased at V2 (p<0.0001) for all comparisons.

To further investigate molecular signature changes involving *DUSP1* and their potential role in PE pathophysiology, we performed a protein pathway analysis (**Figure 5B**). This analysis identified 12 proteins associated with DUSP1. Among them, FLT1 was found, suggesting shared signaling pathways that may explain their similar expression profiles. There were also proteins associated with VEGF (vascular endothelial growth factor) and PGF (placental growth factor), which have previously been described as associated with PE pathophysiology (13)Taken together, these results highlight DUSP1 as a promising blood-based biomarker candidate for the diagnosis of PE in pregnant women.

## Discussion

The clinical diagnosis of PE remains challenging and is often delayed due to the lack of reliable early biomarkers. Although studies have used large biobanks and cohorts, the identification of efficient biomarkers for early PE diagnosis is still warranted. In this study, we adopted a specific study design strategy to investigate new candidate biomarkers by evaluating gene expression at both the blood and placental levels in women with PE—specifically focusing on genes not expressed postpartum, at a distance from delivery. Our study highlights DUSP1 as a promising non-invasive blood biomarker candidate for PE.

Current screening tools are essentially in the form of diagnostic trees combining several risk factors for PE to predict its occurrence in the short term. They combine several early markers: clinical (mean blood pressure), ultrasound (pulsatility index of uterine arteries) and biological (PAPP-A and PlGF), allowing to predict the risk of PE before term—with a false-positive rate of approximately 10%–15% (23, 24). Recent data from literature has opened new avenues through molecular approaches, particularly by exploring gene expression in this pathology (25–27). However, many studies focus on the investigation of genes on samples, at the blood or placenta level, only at the time of diagnosis. The strength of our study was primarily its prospective design, which contributed to its robustness. Controls were rigorously matched to patients with PE based on two major confounding factors: maternal age and gestational age at diagnosis. The two groups (PE and control) were comparable across all baseline characteristics, thereby addressing potential confounding bias. Another strength of our study is its transversality, as patients in each group were followed from the first clinical manifestations of PE through to the postnatal period. Each patient was evaluated at the three major stages of the disease: diagnosis (first symptoms), childbirth (signs of severity requiring fetal delivery and/or maternal intervention), and postpartum (remission). This transversality is a major asset, allowing us to follow the evolution of the PE transcriptional signature in parallel with the progression of the disease.

Our study highlighted a specific gene signature of PE. Among the modulated genes, the associated biological processes have previously been described in the pathophysiology of PE (28, 29). Interestingly, we also identified the *FLT1* gene, whose role as a biomarker in PE is well documented (12, 30). The presence of this gene indicates that the cohort choice and design strategy of the study is similar to previous studies. We showed that *FLT1* has the same significant expression modulation profile as *DUSP1*, with both genes returning to a physiological baseline after pregnancy. We also found that *FLT1* is part of the *DUSP1* pathway. Additional studies based on other cohorts should be carried out to define the relevance of DUSP1 and FLT1 in PE, either as individual biomarker candidates or as part of a combined signature.

Our study identified DUSP1 as a biomarker candidate for PE. DUSP1 belongs to a large superfamily of 30 types of DUSP involved in signal transduction pathways that inactivate mitogen-activated protein (MAP) kinases. Specifically, DUSP1 modulation affects several pathways, including MAP kinase phosphatase activity, tyrosine kinase receptor activity, angiogenesis, and cell–cell signaling (31). Its role in tumor biology is well documented (32).

Interestingly, several studies have also highlighted the relationship between DUSP1 and hypoxia, a major contributor to the placental abnormalities observed in women with PE. Hypoxic conditions lead to *DUSP1* overexpression and increased interaction with hypoxia-inducible factor 1-alpha (HIF-1α), a molecule (33) involved in PE pathogenesis (34, 35). DUSP1 has also been identified as a contributing gene in cases of recurrent miscarriage (36, 37). DUSP1 expression abnormalities in primary human decidual stromal cells or decidua tissue have been linked to the pathophysiology of recurrent miscarriages. Further studies are needed to highlight the mechanism of action of DUSP1 in PE.

Previous studies have investigated DUSP1 as a potential biomarker for the identification of PE (38). The authors investigated *DUSP1* expression in placental tissue and umbilical cord blood. The authors reported conflicting data regarding DUSP1 expression in placental tissue: DUSP1 mRNA expression in the PE group was significantly lower than in the healthy group, whereas protein levels assessed by immunohistochemistry were similar between PE and control groups. Considering DUSP1 as a biomarker, the authors investigated DUSP1 protein levels in umbilical cord blood and found significantly lower DUSP1 expression in PE women compared to healthy donors. Moreover, the authors used a limited cohort (400 controls *versus* 5 PE samples) and did not investigate the gestational age at diagnosis that constitutes a major confounding factor associated with potential confusion bias. In contrast, Yonghong Wang et al. reported an indirect role for DUSP1 in the occurrence of PE (39). The authors reported that miR-141-5p reduced DUSP1 expression *in vitro*, thereby affecting the MAPK/ERK pathway and promoting PE features. Although further studies are needed to identify the role of DUSP1 in PE, this study demonstrated DUSP1 expression in immortalized JEG-3 trophoblastic cells (39), whose role in pregnancy and involvement in PE pathogenesis still need to be defined.

In our prospective study, healthy donors were matched with PE patients based on the two main factors: maternal age and gestational age at diagnosis. To prevent any potential bias, both groups were comparable in all baseline characteristics. From the earliest clinical signs of PE through the postpartum period, patients in each group were monitored. As a result, each patient contributed samples at the three major stages of the disease: diagnosis (first symptoms), delivery (severe signs indicating the need for fetal birth and/or maternal rescue), and postpartum (remission). This transversality is a key advantage for tracking the evolution of the PE transcriptional signature in relation to disease progression.

Our study is limited by the size of the cohort. Validation of DUSP1 as a biomarker candidate for PE should be conducted in larger, multicenter cohorts.

In conclusion, based on an original study design, we report a set of genes associated with PE, some of which have been previously linked to the pathophysiology of the disease. Further investigation of DUSP1 in a larger cohort—both before and after the onset of PE, and including assessments of disease severity—is necessary to confirm its value as a biomarker. The RANSPre study, a French multicenter cohort, may provide an alternative strategy to evaluate this candidate further.

## Data Availability

The datasets presented in this study can be found in online repositories. The names of the repository/repositories and accession number(s) can be found in the article/**Supplementary Material**.

## Supplementary material

Supplementary Figure 1 Study design. Samples from included patients were taken at three major time points: at the diagnosis of preeclampsia (PE) (V1, first symptoms), at childbirth (biopsy from placenta (BP), signs of severity indicating fetal birth and/or maternal rescue), and postpartum (V2, remission). V1 and V2 correspond to whole blood and serum samples; BP corresponds to placental biopsy samples. Total RNA was isolated and analyzed using RNA sequencing(10 samples from PE and control groups). qRTPCR (6 samples from PE and control (C) groups) and ELISA (9 samples from PE and control groups) were performed on isolated RNA and serum samples, respectively. Abbreviations: RNAseq, RNA sequencing; qRT-PCR, quantitative reverse transcription-polymerase chain reaction; ELISA, enzyme-linked immunosorbent assay.

Supplementary Table 1 List of primers used for qRTPCR.

Supplementary Table 2 List of genes associated with the preeclampsia signature.
