# Supplementary figures and images for: Dual specificity phosphatase 1 as a non-invasive circulating biomarker candidate in preeclampsia
Source: Front Endocrinol (Lausanne). 2025 Sep 11;16:1576240. doi: 10.3389/fendo.2025.1576240 (PMC12460085; doi:10.3389/fendo.2025.1576240)

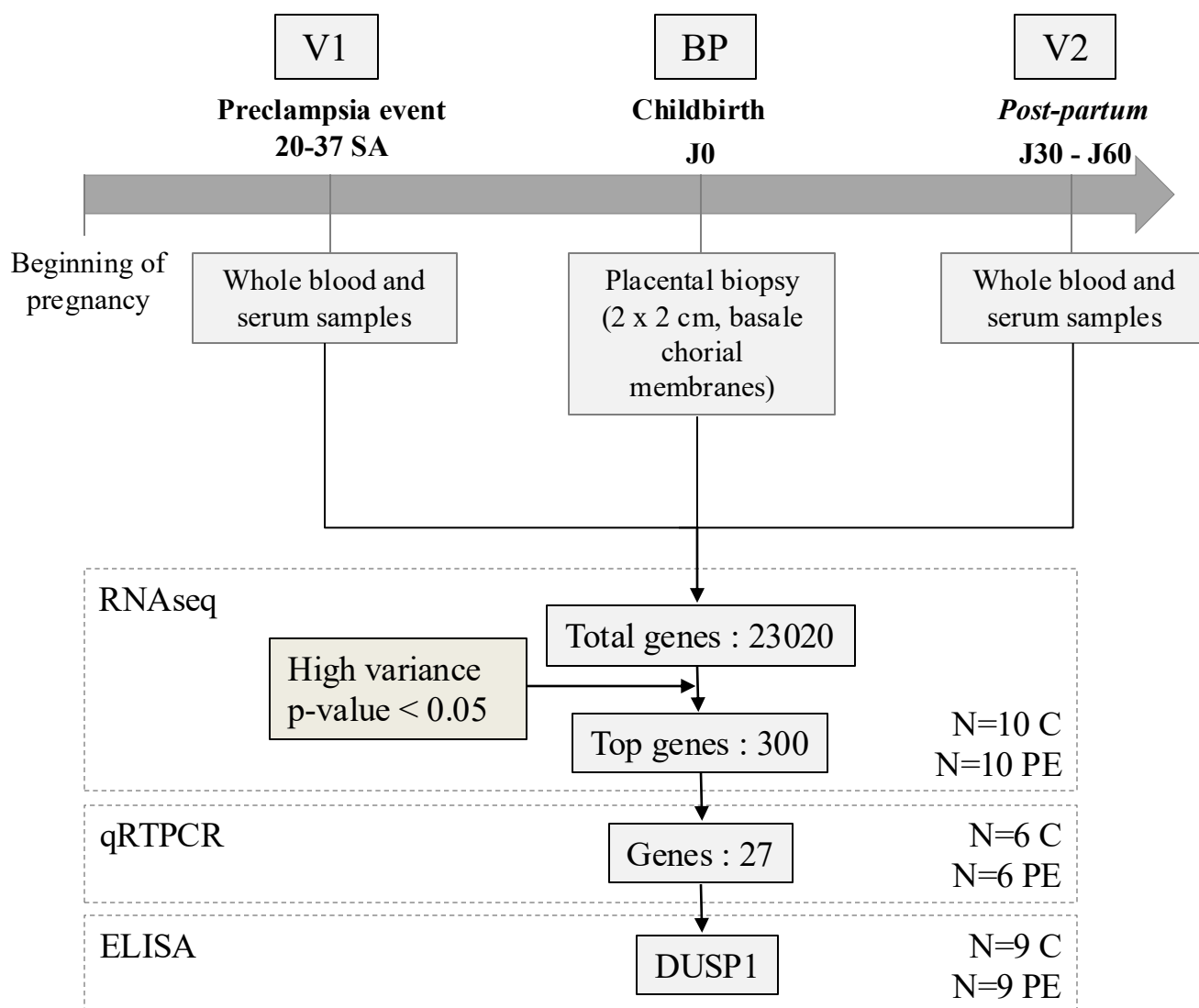

Supplement: Supplementary Figure 1 — Study design. Samples from included patients were taken at three major time points: at the diagnosis of preeclampsia (PE) (V1, first symptoms), at childbirth (biopsy from placenta (BP), signs of severity indicating fetal birth and/or maternal rescue), and postpartum (V2, remission). V1 and V2 correspond to whole blood and serum samples; BP corresponds to placental biopsy samples. Total RNA was isolated and analyzed using RNA sequencing(10 samples from PE and control groups). qRTPCR (6 samples from PE and control (C) groups) and ELISA (9 samples from PE and control groups) were performed on isolated RNA and serum samples, respectively. Abbreviations: RNAseq, RNA sequencing; qRT-PCR, quantitative reverse transcription-polymerase chain reaction; ELISA, enzyme-linked immunosorbent assay. [file Image1.pdf]
