# Supplementary material for: Dual specificity phosphatase 1 as a non-invasive circulating biomarker candidate in preeclampsia
Source: Front Endocrinol (Lausanne). 2025 Sep 11;16:1576240. doi: 10.3389/fendo.2025.1576240 (PMC12460085; doi:10.3389/fendo.2025.1576240)
Supplement: Supplementary Table 1 — List of primers used for qRTPCR. [file Table1.docx]

**Table S1 List of primers used for qRTPCR.**

| **Gene** | **Forward primer (5'-3')** | **Reverse primer (5'-3')** |
| --- | --- | --- |
| *BIN2* | GGCTGCTATGTGACCATCTTCC | GCTTCTCCAGTTTGCTCATCACC |
| *IRS2* | CCTGCCCCCTGCCAACACCT | TGTGACATCCTGGTGATAAAGCC |
| *STAG3* | CTGAAACGCCTCTCTGCCTTCT | GAGTCAAGGCTGGCAGGATAAC |
| *ARMC12* | CAAAAGCGTAGTCAGCCTGG | GATGCAGATGGGTGAGTTGC |
| *PER1* | TCAACTGCCTGGACAGCATCCT | TCAGAGGCTGAGGAGGTGGTAT |
| *ANG* | CAACAGTGTCCTTCAGAAGCAGC | CCAGCTTGATATACATCTGCACAG |
| *DUSP1* | CAACCACAAGGCAGACATCAGC | GTAAGCAAGGCAGATGGTGGCT |
| *FSTL1* | TCGCATCATCCAGTGGCTGGAA | TCACTGGAGTCCAGGCGAGAAT |
| *TBXA2R* | CCTGGGTGTATATCCTGTTCCG | AGATCGTGCCACTGTACTCCAG |
| *TCN2* | CAGAACAGTGCGAGAGGAGATC | TCGCCTTGAGACATGCTGTTCC |
| *OASL* | GTGCCTGAAACAGGACTGTTGC | CCTCTGCTCCACTGTCAAGTGG |
| *CD163* | CCAGAAGGAACTTGTAGCCACAG | CAGGCACCAAGCGTTTTGAGCT |
| *GPER1* | TTCCGCGAGAAGATGACCATCC | TAGTACCGCTCGTGCAGGTTGA |
| *ITGA2B* | CTGTCCAGCTACTGGTGCAAGA | ATGTTGTGCCCAGTGGCTCCAA |
| *THBS1* | GCTGGAAATGTGGTGCTTGTCC | CTCCATTGTGGTTGAAGCAGGC |
| *SPAG6* | GACAGTAGTGGATGCAGGAGCT | CCATTTCTGCCAGATCCACGGA |
| *A2M* | TTGAAGAGCCTCACACGGAGA | TTCCACTCGGTGATGGTGTCAG |
| *FN1* | ACAACACCGAGGTGACTGAGAC | GGACACAACGATGCTTCCTGAG |
| *FLT1* | CCTGCAAGATTCAGGCACCTATG | GTTTCGCAGGAGGTATGGTGCT |
| *ADAMTS2* | TACAAGGACGCCTTCAGCCTCT | CCACTTTGCAGTGGCTGTTGTC |
| *SDK1* | GGACACATCTCTCAAGGTCAGC | TGTGCGTGAGACGAGAGTCGTT |
| *EPCAM* | GCCAGTGTACTTCAGTTGGTGC | CCCTTCAGGTTTTGCTCTTCTCC |
| *ESRG* | ATGAAAGGGAAGACATACAA | TGAACATAGCAAGGGAAA |
| *TNFRSF21* | CCAGTGCCATTGTGGAAAAGGC | CTTCCCACTTGGGCTGCTACAA |
| *PTPRS* | CTCGCCCAAGAACTTCAAGGTG | AGGTGCGTGATGAGCTTCTTGG |
| *NRP1* | AACAACGGCTCGGACTGGAAGA | GGTAGATCCTGATGAATCGCGTG |
| *MIR25* | CGGAGACTTGGGCAATT | GAACATGTCTGCGTATCTC |
| *GRB10* | AGGACACAGCACTGGTTTCACG | TCTGGCTGTCACGGAGGAGAAA |
| *ACTB* | GGAAATCGTGCGTGACATTA | AGGAGGAAGGCTGGAAGAG |

**Table S2 List of genes associated to the preeclampsia signature.**

| **Clusters** | **Gene name** | **Complete name** |
| --- | --- | --- |
| **Angiogenesis, Differentiation** | *ARMC12* | Armadillo repeat containing 12 |
|  | *ANG* | Angiogenin |
|  | *FSTL1* | Follistatin like 1 |
|  | *DUSP1* | Dual specificity phosphatase |
|  | *FN1* | Fibronectin |
|  | *FLT1* | FMS related receptor tyrosine kinase 1 |
|  | *NRP1* | Neuropilin 1 |
|  | *GRB10* | Growth factor receptor bound protein 10 |
| **Cell adhesion** | *ITGA2B* | Integrin subunit alpha 2b |
|  | *THBS1* | Thrombospondin |
|  | *SDK1* | Sidekick cell adhesion molecule 1 |
|  | *EPCAM* | Epithelial cell adhesion molecule |
| **Cell cycle** | *IRS2* | Insulin receptor substrate 2 |
|  | *STAG3* | Stromal antigen 3 |
|  | *MIR25* | Micro RNA 25 |
|  | *ESRG* | Embryos stem cell related |
|  | *GPER1* | G protein coupled estrogen receptor 1 |
|  | *PER1* | Period circadian regulator 1 |
|  | *BIN2* | Bridging integrator 2 |
| **Inflammatory response** | *CD163* | Cluster differentiation 163 |
|  | *TNFRSF21* | TNF receptor superfamily member 21 |
| **Cell metabolism** | *TCN2* | Transcobalamin 2 |
|  | *SPAG6* | Sperm associated antigen 6 |
|  | *A2M* | Alpha 2 macrobulin |
|  | *ADAMTS2* | ADAM metallopeptidase with thrombospondin type 1 motif 2 |
